# Crystal structure of 1-butyl-3-{2-[(indan-5-yl)amino]-2-oxoeth­yl}-1*H*-imidazol-3-ium chloride

**DOI:** 10.1107/S2056989018014792

**Published:** 2018-10-26

**Authors:** Vidya Zende, Tejpalsingh Ramsingh Girase, Nicolas Chrysochos, Anant Ramakant Kapdi, Carola Schulzke

**Affiliations:** a Department of Chemistry, Institute of Chemical Technology, Nathalal Parekh Road, Matunga, Mumbai 400019, India; b Institut für Biochemie, Universität Greifswald, Felix-Hausdorff-Strasse 4, 17487 Greifswald, Germany

**Keywords:** crystal structure, *N*-heterocyclic carbenes, imidazolium salt, amido-functionalization, electron-rich ligand, sigma donor acetamide, hydrogen bonding

## Abstract

In the cation of the title mol­ecular salt, C_18_H_24_N_3_O^+^·Cl^−^, an intra­molecular C—H⋯O hydrogen bond stabilizes the almost coplanar orientation of the aromatic ring of the indane unit and the amide plane. In the crystal, the packing is dominated by inter­molecular C—H⋯Cl hydrogen-bonding inter­actions that result in the formation of slab-like structures propagating along [010]. The slabs are linked by weak C—H⋯O inter­actions, forming layers lying parallel to (100). The methyl­ene carbon atom of the indanyl substituent is disordered over two positions with a refined occupancy ratio of 0.84 (2):0.16 (2). The crystal studied was refined as a twin with matrix [1 0 0.9, 0  0, 0 0 ]; the resulting BASF value is 0.30.

## Abstract 2

A new amide moiety bearing an imidazolium salt as precursor to an *N*-heterocyclic carbene was synthesized. The synthetic procedure, the compound’s characterization and its crystal structure, including a comparison of geometrical parameters with related compounds, are reported and discussed.

## Chemical context   

*N*-Heterocyclic carbenes (NHCs) are neutral compounds in which a 6e^−^-containing divalent carbon atom is placed between two hetero atoms. They are typically derived from their parent imidazolium salts by deprotonation of the carbon atom located in between the two nitro­gen atoms (Bhatia *et al.*, 2013 ▸). The high reactivity in the case of carbenes can be attributed to the presence of an incomplete octet resulting in a strong electron-donating ability (Hopkinson *et al.*, 2014 ▸). Arduengo was the first to successfully isolate a free carbene and characterize it by obtaining a single crystal X-ray structure for the same. This study opened a new era in organic chemistry allowing the investigation of the so-called NHCs as ligands (Arduengo *et al.*, 1991 ▸). To date, a tremendous amount of research on NHCs has enhanced the popularity of these carbene compounds in organic synthesis, organometallic chemistry, organocatalysis, medicinal and pharmaceutical applications and essentially every discipline of modern day science. Over the past two decades, *N*-heterocyclic carbene (NHC) ligands have been among the most exploited in organic synthesis. They can be considered superior to phosphine ligands as their electronic and steric properties can be easily fine-tuned by simple variations in their structures (Díez-González *et al.*, 2009 ▸; Hermann, 2002 ▸; Froese *et al.*, 2017 ▸). Attempts have been made to tune or modify the electronic and steric properties of NHCs by changing the substituent at one or both nitro­gen centres. These changes in electronics and steric properties may further provide subtle information about the mechanism of catalytic transformations (Huynh, 2018 ▸; Peris, 2018 ▸). Although the term hemilability for a coordinated ligand was first introduced in 1979 (Jeffrey & Rauchfuss, 1979 ▸), the first hemilabile NHC ligand was developed some twenty years later (McGuinness & Cavell, 2000 ▸). The presence of hemilabile coordination sites in a ligand system plays a crucial role in catalysis as well as in biological sciences (cytotoxicity). The modular electronic and steric properties of the hemilabile ligand systems provide extra stability to transition metal complexes (Peris, 2018 ▸; Normand & Cavell, 2008 ▸). Herein we present the synthesis and crystal structure of the chloride salt of the potentially hemilabile amido-functionalized NHC ligand precursor, 1-butyl-3-{2-[(indan-5-yl)amino]-2-oxoeth­yl}-1*H*-imidazol-3-ium.
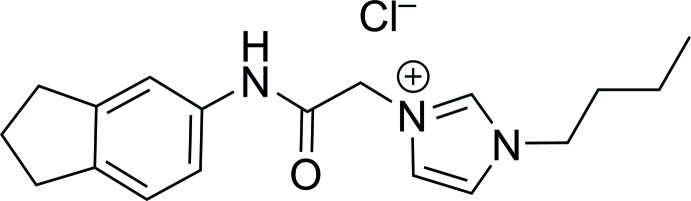


## Structural commentary   

The title compound, consists of a chloride anion and an N-substituted imidazolium cation, combining the NHC precursor moiety with a amide (–NH–C(O)–CH_2_–) moiety. The amide group is linked to one nitro­gen of the imidazolium ring, N2, by a methyl­ene group, and bears on its opposite side a indanyl substituent bound to the amide nitro­gen atom N1. The other (non-amidic) substituent on the second nitro­gen atom, N3, of the imidazolium ring is an extended *n*-butyl chain, whose mean plane (C15–C18) is inclined to the plane of the imidazolium ring (N2/N3/C12–C14) by 73.2 (6)°. The central –CH_2_– C atom, C1, of the indanyl substituent is disordered over two positions with a refined occupancy ratio of C1:C1′ = 0.84 (2):0.16 (2). Atom C1 resides 0.393 (12) Å below the plane (on the opposite side of the imidazolium moiety) of the four planar C atoms of the pentene ring (C2–C5), while atom C1′ is 0.40 (4) Å above this plane (*i.e.* on the same side as the imidazolium moiety).

**Figure 1:**
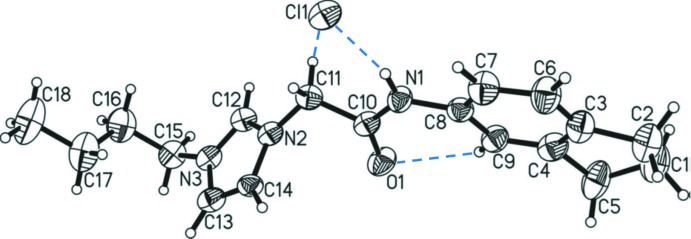
Mol­ecular structure of the title mol­ecular salt, with the atom labelling and displacement ellipsoids drawn at the 50% probability level. In this and subsequent figures, only the major component of the disordered atom C1 is shown. The hydrogen bonds (Table 1 ▸) are shown as blue dashed lines.

Crystallographic data of NHC precursor cations substituted by CH_2_–C(O)–NH functional groups (amides) are relatively scarce. A search of the Cambridge Structural Database (CSD, version 3.59, August 2018; Groom *et al.*, 2016 ▸) yielded only 16 hits. Compared to published values of imidazolium salts with amide substituents, the geometrical parameters of the title compound are decidedly unexceptional, falling within the reported ranges. Only the C—C bond between methyl­ene atom C11 and the carbonyl carbon C10 is relatively short [C10—C11 = 1.506 (6) Å] and thereby close to the shortest such bond reported to date, *i.e*. 1.502 Å for a related compound with no substituent on the amide and a dodecyl chain on the other side of the imidazolium cation (Lee *et al.*, 2003*a* ▸). In general, all bond lengths of the two moieties and the methyl­ene linker are in rather close ranges with the largest differences observed being those which lead to further substituents. These are the N—C bond of the amide to its substituent on N ranging from *ca* 1.409 Å for a phenyl (Samantaray *et al.*, 2007 ▸) to 1.482 Å for a *t-*butyl (Ray *et al.*, 2007 ▸), and the N—C bond of the imidazolium ring to the second substituent ranging from *ca* 1.422 Å for a pyrimidyl (Lee *et al.*, 2009 ▸) to 1.483 Å for a rather bulky 3,5-di-*tert*-butyl-2-hy­droxy­benzyl (Wan & Zhang, 2016 ▸). Given the variety of the substituents on both sides in published structures, this observation is not surprising as the potential extension of the π system beyond the imidazolium and amide moieties would be expected to have a considerable influence on these bond lengths. Strictly within the imidazolium and amide moieties, the strongest deviation is found for the amide C(O)—N bond [here C10—N1 = 1.339 (6) Å] ranging from *ca* 1.301 Å for an unsubstituted amide, *i.e.* –C(O)–NH_2_, (Lee *et al.*, 2003*b* ▸) to 1.355 Å for a phenyl-substituted amide (Lee & Zeng, 2012 ▸). A shorter C10—N1 bond is indicative of a strong tautomeric effect, *i.e*. C=O double-bond delocalization towards a C=N double bond. In the title compound, the nitro­gen atom of the amide is bound to an indanyl group and the C10—N1 bond length of 1.339 (6) Å comprises a rather average value for a –C(O)–NH– bond. A value that often varies in such compounds is the angle at which the plane of the amide moiety [–CH_2_–C(O)–NH, calculated without H-atom positions] is arranged with respect to the imidazolium ring plane (C_3_N_2_). Here the dihedral angles range from *ca* 42.64° (Lee *et al.*, 2003*b* ▸) to 85.95° (Lee *et al.*, 2012 ▸). In the title compound, this dihedral angle is 71.9 (3)°. At this angle, resonance between the two moieties (amide and imidazolium) can clearly be excluded. In contrast, the angle between the amide moiety and the aromatic ring of the indanyl substituent is only 18.1 (2)°, suggesting together with the N1—C8 bond length of only 1.427 (6) Å, that the resonance of the aromatic ring extends to the amide moiety and/or *vice versa*. This relative orientation of the two aromatic systems is probably supported by a weak intra­molecular C—H⋯O hydrogen bond, between the amide oxygen atom (O1) and the aromatic carbon atom C9 (Table 1 ▸ and Fig. 1 ▸).

## Supra­molecular features   

**Figure 2:**
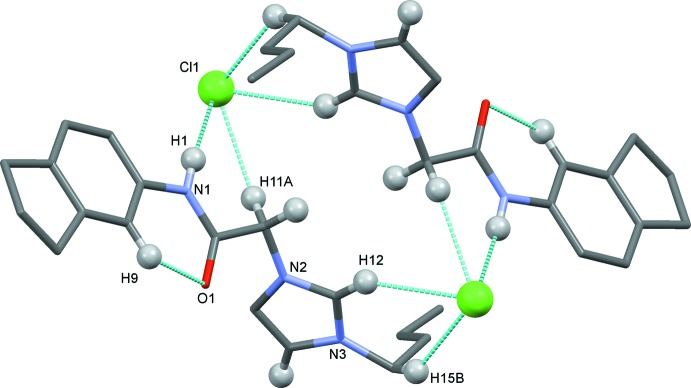
A view of the two-by-two hydrogen-bonded unit (dashed lines; see Table 1 ▸ for details). Only the H atoms (grey balls) involved in the intra- and inter­molecular inter­actions have been included. The unlabelled atoms are related to the labelled atoms by the symmetry operation −*x* + 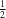, −*y* + 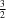, −*z*.

**Figure 3:**
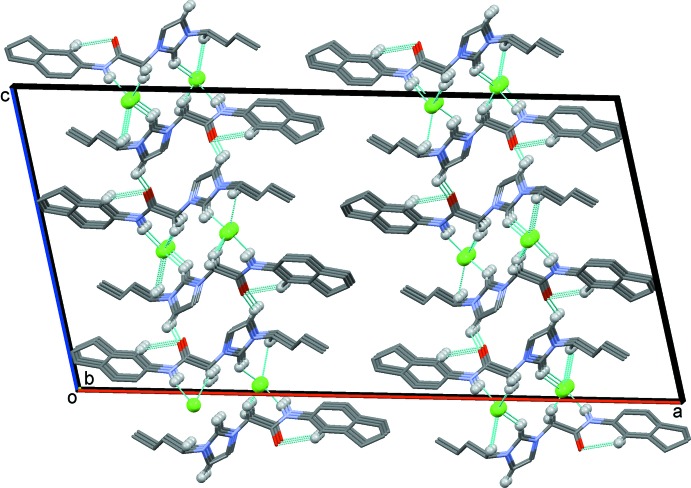
Crystal packing of the title mol­ecular salt, viewed along the *b* axis, showing the various hydrogen bonds as dashed lines (see Table 1 ▸ for details). Only the H atoms (grey balls) involved in these inter­actions have been included.

The comparably large unit cell of the crystal structure with *Z* = 8 is rather thin with a short *b* axis of 5.3986 (11) Å, and the eight imidazolium cations are arranged in a single layer within the cell. The chloride anions and imidazolium cations form symmetric pairs, two-by-two, supported by C—H⋯Cl hydrogen bonds involving hydrogen atoms of an *n*-butyl methyl­ene C atom (C15—H15*B*), an imidazolium C atom (C12—H12), the amide N atom (N1—H1) and a C atom of the methyl­ene linker (C11—H11*A*), and the chloride anion Cl1 (Table 1 ▸ and Fig. 2 ▸). In the crystal, these two-by-two units are linked by C11—H11*B*⋯Cl1^ii^ hydrogen bonds, forming slab-like structures propagating along the *b*-axis direction (Table 1 ▸ and Fig. 3 ▸). Weak C13—H13⋯O1^iii^ inter­actions link the slabs to form layers lying parallel to the *bc* plane (Table 1 ▸ and Fig. 3 ▸)

## Synthesis and crystallization   

The title compound, was synthesized by the simple reaction of *n*-butyl imidazole with 2-chloro-*N*-(indan-5-yl)acetamide in dry aceto­nitrile as solvent. All reagents and solvents required for the synthesis were purchased commercially and used without any further purification.

**Synthesis of 1-butyl-3-{2-[(indan-5-yl)amino]-2-oxoeth­yl}-1** ***H*** **-imidazol-3-ium chloride:** The synthesis of the imidazolium salt was carried out under a nitro­gen atmosphere. An oven-dried Schlenk tube was charged with a stirring bar, 1.00 mmol of 2-chloro-*N*-(indan-5-yl)acetamide, 1.5 mmol of *n*-butyl imidazole, and 2 ml of dry aceto­nitrile. The reaction mixture was stirred for 12 h at 353 K. After the reaction mixture was allowed to cool to r.t., diethyl ether was added to the reaction mixture upon which the product precipitated leading already to sufficient separation. The precipitate was isolated by carefully deca­nting off the solvent, then washed with acetone (2 × 5ml) and hexane (2 × 5 ml), and dried under vacuum. The product was obtained as a colourless (white) solid; yield: 94%. Colourless prismatic crystals suitable for X-ray diffraction analysis were obtained by slow evaporation of a solution in ethanol.

^1^H NMR (400 MHz, DMSO-*d*_6_): δ 10.99 (*s*, 1H), 9.26 (*s*, 1H), 7.79 (*d*, *J* = 10.5 Hz, 2H), 7.50 (*s*, 1H), 7.34 (*d*, *J* = 9.1 Hz, 1H), 7.11 (*d*, *J* = 8.1 Hz, 1H), 5.24 (*s*, 2H), 4.20 (*t*, *J* = 7.1 Hz, 2H), 2.77 (*q*, *J* = 7.7 Hz, 4H), 1.95 (*qui*, *J* = 7.4 Hz, 2H), 1.79–1.70 (*m*, 2H), 1.28–1.18 (*m*, 2H), 0.87 (*t*, *J* = 7.4 Hz, 3H). ^13^C NMR (101 MHz, DMSO-*d*_6_): δ 163.8, 144.6, 139.3, 137.8, 137.1, 124.7, 124.4, 122.1, 117.6, 115.7, 51.7, 49.0, 32.9, 32.1, 31.7, 25.5, 19.1, 13.7. Analysis calculated for C_17_H_24_ClN_3_O: C, 64.76; H, 7.25; N, 12.59. Found: C, 64.59; H, 7.12; N, 12.68. IR: C=O Stretching 1700.23 cm^−1^.

## Refinement   

Crystal data, data collection and structure refinement details are summarized in Table 2 ▸. The N-bound hydrogen atom (H1) was located in a difference-Fourier map and freely refined. The C-bound H atoms were placed in calculated positions and treated as riding: C—H = 0.95-0.99 Å with *U*_iso_(H) = 1.5*U*_eq_(C-meth­yl) and 1.2*U*_eq_(C) for other H atoms.

The methyl­ene carbon atom C1 of the indanyl substituent is disordered over two positions with a refined occupancy ratio of 0.84 (2):0.16 (2). This disorder was modelled with constraints (SADI for all C-C bonds involving C1, SIMU and DELU). The crystal studied was refined as a twin with matrix [1 0 0.9, 0 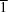 0, 0 0 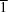]; the resulting BASF value is 0.30.

## Supplementary Material

Crystal structure: contains datablock(s) I, Global. DOI: 10.1107/S2056989018014792/su5456sup1.cif

Structure factors: contains datablock(s) I. DOI: 10.1107/S2056989018014792/su5456Isup2.hkl

CCDC reference: 1832439

Additional supporting information:  crystallographic information; 3D view; checkCIF report

## Figures and Tables

**Table 1:** Hydrogen-bond geometry (Å, °)

*D*—H⋯*A*	*D*—H	H⋯*A*	*D*⋯*A*	*D*—H⋯*A*
C9—H9⋯O1	0.95	2.35	2.915 (6)	118
N1—H1⋯Cl1	0.87 (5)	2.32 (5)	3.177 (4)	167 (4)
C11—H11*A*⋯Cl1	0.99	2.75	3.541 (4)	137
C12—H12⋯Cl1^i^	0.95	2.73	3.530 (4)	143
C15—H15*B*⋯Cl1^i^	0.99	2.71	3.493 (5)	136
C11—H11*B*⋯Cl1^ii^	0.99	2.54	3.432 (5)	150
C13—H13⋯O1^iii^	0.95	2.54	3.066 (5)	115

Symmetry codes: (i) 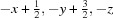; (ii) 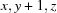; (iii) 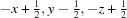.

**Table 2:** Experimental details

Crystal data	
Chemical formula	C_18_H_24_N_3_O^+^·Cl^−^
*M*_r_	333.85
Crystal system, space group	Monoclinic, *C*2/*c*
Temperature (K)	170
*a*, *b*, *c* (Å)	36.270 (7), 5.3986 (11), 18.620 (4)
β (°)	103.34 (3)
*V* (Å^3^)	3547.6 (13)
*Z*	8
Radiation type	Mo *K*α
μ (mm^−1^)	0.22
Crystal size (mm)	0.35 × 0.27 × 0.13

Data collection	
Diffractometer	Stoe IPDS2T
Absorption correction	Numerical (*X-RED32* and *X-SHAPE*; Stoe & Cie, 2010 ▸)
*T*_min_, *T*_max_	0.561, 0.968
No. of measured, independent and observed [*I* > 2σ(*I*)] reflections	12392, 3134, 1953
*R*_int_	0.119
(sin θ/λ)_max_ (Å^−1^)	0.595

Refinement	
*R*[*F* ^2^ > 2σ(*F* ^2^)], *wR*(*F* ^2^), *S*	0.066, 0.199, 1.05
No. of reflections	3134
No. of parameters	224
No. of restraints	36
H-atom treatment	H atoms treated by a mixture of independent and constrained refinement
Δρ_max_, Δρ_min_ (e Å^−3^)	0.44, −0.40

Computer programs: *X-AREA* (Stoe & Cie, 2010 ▸), *X-RED32* (Stoe & Cie, 2010 ▸), *SHELXT2018* (Sheldrick, 2015*a* ▸), *XP* (Sheldrick, 2008 ▸), *Mercury* (Macrae *et al.*, 2008 ▸), *SHELXL2018* (Sheldrick, 2015*b* ▸), *WinGX* (Farrugia, 2012 ▸), *PLATON* (Spek, 2009 ▸) and *publCIF* (Westrip, 2010 ▸).

## supplementary crystallographic information

### Crystal data

**Table:**

C_18_H_24_N_3_O^+^·Cl^−^	*F*(000) = 1424
*M**_r_* = 333.85	*D*_x_ = 1.250 Mg m^−^^3^
Monoclinic, *C*2/*c*	Mo *K*α radiation, λ = 0.71073 Å
*a* = 36.270 (7) Å	Cell parameters from 19866 reflections
*b* = 5.3986 (11) Å	θ = 6.6–59.3°
*c* = 18.620 (4) Å	µ = 0.22 mm^−^^1^
β = 103.34 (3)°	*T* = 170 K
*V* = 3547.6 (13) Å^3^	Prism, colourless
*Z* = 8	0.35 × 0.27 × 0.13 mm

### Data collection

**Table:**

Stoe IPDS2T diffractometer	3134 independent reflections
Radiation source: fine-focus sealed tube	1953 reflections with *I* > 2σ(*I*)
Detector resolution: 6.67 pixels mm^-1^	*R*_int_ = 0.119
ω scans	θ_max_ = 25.0°, θ_min_ = 3.5°
Absorption correction: numerical (X-Red32 and X-Shape; Stoe & Cie, 2010)	*h* = −37→42
*T*_min_ = 0.561, *T*_max_ = 0.968	*k* = −6→6
12392 measured reflections	*l* = −21→22

### Refinement

**Table:**

Refinement on *F*^2^	Primary atom site location: structure-invariant direct methods
Least-squares matrix: full	Secondary atom site location: difference Fourier map
*R*[*F*^2^ > 2σ(*F*^2^)] = 0.066	Hydrogen site location: mixed
*wR*(*F*^2^) = 0.199	H atoms treated by a mixture of independent and constrained refinement
*S* = 1.05	*w* = 1/[σ^2^(*F*_o_^2^) + (0.1126*P*)^2^ + 0.3489*P*] where *P* = (*F*_o_^2^ + 2*F*_c_^2^)/3
3134 reflections	(Δ/σ)_max_ < 0.001
224 parameters	Δρ_max_ = 0.44 e Å^−^^3^
36 restraints	Δρ_min_ = −0.40 e Å^−^^3^

### Special details

**Table:**

Geometry. All esds (except the esd in the dihedral angle between two l.s. planes) are estimated using the full covariance matrix. The cell esds are taken into account individually in the estimation of esds in distances, angles and torsion angles; correlations between esds in cell parameters are only used when they are defined by crystal symmetry. An approximate (isotropic) treatment of cell esds is used for estimating esds involving l.s. planes.
Refinement. Refined as a 2-component twin.

### Fractional atomic coordinates and isotropic or equivalent isotropic displacement parameters (Å^2^)

**Table:**

	*x*	*y*	*z*	*U*_iso_*/*U*_eq_	Occ. (<1)
N1	0.16282 (10)	0.4778 (7)	0.0721 (2)	0.0366 (9)	
N2	0.25866 (10)	0.7006 (6)	0.13822 (19)	0.0336 (8)	
N3	0.31102 (11)	0.8875 (6)	0.1857 (2)	0.0358 (8)	
O1	0.18710 (10)	0.7509 (6)	0.16419 (18)	0.0477 (8)	
C1	0.0069 (2)	0.6310 (17)	0.1297 (7)	0.065 (3)	0.84 (2)
H1A	−0.005777	0.746242	0.090345	0.078*	0.84 (2)
H1B	−0.008010	0.623539	0.167931	0.078*	0.84 (2)
C1'	0.0140 (11)	0.538 (7)	0.163 (2)	0.056 (9)	0.16 (2)
H1'1	0.019558	0.439312	0.209509	0.067*	0.16 (2)
H1'2	−0.009825	0.630514	0.160560	0.067*	0.16 (2)
C2	0.01033 (15)	0.3703 (9)	0.0973 (4)	0.0588 (15)	
H2A	0.007424	0.238929	0.132728	0.071*	
H2B	−0.008830	0.345562	0.050560	0.071*	
C3	0.05004 (14)	0.3727 (9)	0.0845 (3)	0.0479 (12)	
C4	0.07030 (15)	0.5732 (8)	0.1194 (3)	0.0464 (12)	
C5	0.04634 (15)	0.7158 (10)	0.1626 (4)	0.0629 (16)	
H5A	0.053825	0.675188	0.215787	0.075*	
H5B	0.048800	0.896697	0.156268	0.075*	
C6	0.06731 (15)	0.2103 (10)	0.0450 (3)	0.0539 (13)	
H6	0.053560	0.074806	0.019341	0.065*	
C7	0.10502 (14)	0.2478 (8)	0.0434 (3)	0.0489 (12)	
H7	0.117384	0.133484	0.018010	0.059*	
C8	0.12457 (12)	0.4493 (8)	0.0782 (2)	0.0361 (10)	
C9	0.10734 (13)	0.6165 (8)	0.1167 (3)	0.0404 (10)	
H9	0.120702	0.756550	0.140374	0.048*	
C10	0.19013 (13)	0.6220 (7)	0.1116 (2)	0.0351 (10)	
C11	0.22575 (12)	0.6170 (8)	0.0831 (2)	0.0366 (10)	
H11A	0.230239	0.446019	0.067916	0.044*	
H11B	0.222334	0.724566	0.038996	0.044*	
C12	0.28154 (12)	0.8823 (7)	0.1286 (2)	0.0337 (10)	
H12	0.277478	0.991130	0.087397	0.040*	
C13	0.30716 (14)	0.7024 (8)	0.2339 (3)	0.0404 (11)	
H13	0.324273	0.664753	0.279459	0.048*	
C14	0.27447 (12)	0.5842 (7)	0.2044 (2)	0.0346 (10)	
H14	0.264207	0.446855	0.225054	0.042*	
C15	0.34367 (14)	1.0541 (8)	0.1924 (3)	0.0448 (11)	
H15A	0.355727	1.079649	0.245245	0.054*	
H15B	0.334866	1.217060	0.170671	0.054*	
C16	0.37271 (15)	0.9480 (10)	0.1533 (4)	0.0598 (15)	
H16A	0.359918	0.915740	0.101139	0.072*	
H16B	0.392405	1.075129	0.153460	0.072*	
C17	0.39176 (17)	0.7154 (11)	0.1855 (4)	0.0678 (17)	
H17A	0.372720	0.581383	0.180888	0.081*	
H17B	0.403047	0.741053	0.238706	0.081*	
C18	0.42267 (19)	0.6376 (13)	0.1468 (5)	0.089 (2)	
H18A	0.411994	0.626255	0.093572	0.133*	
H18B	0.432805	0.475814	0.165602	0.133*	
H18C	0.443086	0.760623	0.156499	0.133*	
H1	0.1675 (12)	0.378 (8)	0.039 (3)	0.029 (11)*	
Cl1	0.19326 (3)	0.10403 (18)	−0.03275 (7)	0.0429 (3)	

### Atomic displacement parameters (Å^2^)

**Table:**

	*U*^11^	*U*^22^	*U*^33^	*U*^12^	*U*^13^	*U*^23^
N1	0.036 (2)	0.037 (2)	0.038 (2)	0.0015 (16)	0.0122 (17)	−0.0060 (18)
N2	0.042 (2)	0.0261 (17)	0.035 (2)	0.0016 (15)	0.0149 (17)	−0.0008 (15)
N3	0.044 (2)	0.0271 (17)	0.040 (2)	−0.0053 (16)	0.0163 (17)	−0.0018 (15)
O1	0.052 (2)	0.0487 (18)	0.047 (2)	−0.0017 (15)	0.0207 (16)	−0.0168 (16)
C1	0.052 (4)	0.058 (5)	0.091 (7)	0.015 (3)	0.027 (4)	−0.003 (5)
C1'	0.043 (15)	0.046 (17)	0.091 (19)	0.016 (14)	0.042 (14)	0.001 (15)
C2	0.049 (3)	0.054 (3)	0.079 (4)	0.001 (2)	0.026 (3)	0.003 (3)
C3	0.044 (3)	0.046 (3)	0.056 (3)	0.002 (2)	0.016 (2)	0.003 (2)
C4	0.055 (3)	0.041 (2)	0.046 (3)	0.009 (2)	0.018 (2)	0.002 (2)
C5	0.055 (3)	0.053 (3)	0.091 (4)	0.010 (3)	0.037 (3)	−0.002 (3)
C6	0.047 (3)	0.048 (3)	0.069 (4)	−0.004 (2)	0.017 (3)	−0.009 (3)
C7	0.051 (3)	0.040 (3)	0.057 (3)	0.000 (2)	0.015 (3)	−0.010 (2)
C8	0.040 (2)	0.036 (2)	0.033 (2)	0.0031 (18)	0.0089 (19)	0.0037 (18)
C9	0.044 (3)	0.037 (2)	0.042 (3)	0.004 (2)	0.014 (2)	−0.001 (2)
C10	0.044 (3)	0.028 (2)	0.036 (2)	0.0026 (19)	0.015 (2)	−0.0012 (19)
C11	0.042 (2)	0.032 (2)	0.036 (2)	−0.0015 (19)	0.0079 (19)	−0.0058 (19)
C12	0.045 (3)	0.0210 (19)	0.037 (2)	−0.0004 (18)	0.012 (2)	−0.0011 (17)
C13	0.054 (3)	0.031 (2)	0.040 (3)	0.003 (2)	0.018 (2)	0.0033 (19)
C14	0.043 (2)	0.031 (2)	0.031 (2)	0.0051 (19)	0.0115 (19)	0.0088 (18)
C15	0.050 (3)	0.033 (2)	0.053 (3)	−0.008 (2)	0.014 (2)	−0.003 (2)
C16	0.052 (3)	0.051 (3)	0.079 (4)	−0.014 (2)	0.019 (3)	−0.003 (3)
C17	0.060 (4)	0.051 (3)	0.097 (5)	−0.005 (3)	0.027 (3)	−0.014 (3)
C18	0.071 (4)	0.074 (4)	0.136 (7)	−0.005 (3)	0.055 (5)	−0.026 (4)
Cl1	0.0575 (7)	0.0305 (5)	0.0449 (7)	0.0024 (5)	0.0200 (5)	−0.0028 (5)

### Geometric parameters (Å, º)

**Table:**

N1—C10	1.339 (6)	C2—C3	1.514 (7)
N1—C8	1.427 (6)	C3—C4	1.383 (7)
N2—C12	1.324 (5)	C3—C6	1.384 (7)
N2—C14	1.384 (5)	C4—C9	1.376 (7)
N2—C11	1.455 (6)	C4—C5	1.522 (7)
N3—C12	1.323 (6)	C6—C7	1.390 (7)
N3—C13	1.372 (5)	C7—C8	1.376 (6)
N3—C15	1.469 (6)	C8—C9	1.388 (6)
O1—C10	1.227 (5)	C10—C11	1.506 (6)
C1—C5	1.491 (8)	C13—C14	1.347 (7)
C1—C2	1.547 (8)	C15—C16	1.524 (7)
C1'—C2	1.508 (16)	C16—C17	1.491 (8)
C1'—C5	1.518 (16)	C17—C18	1.525 (8)
			
C10—N1—C8	129.0 (4)	C1'—C5—C4	102.8 (11)
C12—N2—C14	108.3 (4)	C3—C6—C7	119.2 (5)
C12—N2—C11	124.9 (4)	C8—C7—C6	120.5 (4)
C14—N2—C11	126.2 (3)	C7—C8—C9	120.9 (4)
C12—N3—C13	109.0 (4)	C7—C8—N1	116.9 (4)
C12—N3—C15	124.5 (4)	C9—C8—N1	122.2 (4)
C13—N3—C15	126.3 (4)	C4—C9—C8	117.9 (4)
C5—C1—C2	106.5 (5)	O1—C10—N1	125.3 (4)
C2—C1'—C5	107.1 (13)	O1—C10—C11	122.2 (4)
C1'—C2—C3	102.4 (11)	N1—C10—C11	112.5 (4)
C3—C2—C1	102.6 (5)	N2—C11—C10	112.1 (4)
C4—C3—C6	119.3 (5)	N3—C12—N2	108.8 (4)
C4—C3—C2	110.8 (4)	C14—C13—N3	106.9 (4)
C6—C3—C2	130.0 (5)	C13—C14—N2	107.1 (4)
C9—C4—C3	122.2 (4)	N3—C15—C16	111.2 (4)
C9—C4—C5	128.0 (5)	C17—C16—C15	115.5 (5)
C3—C4—C5	109.7 (4)	C16—C17—C18	111.4 (6)
C1—C5—C4	103.8 (5)		

### Hydrogen-bond geometry (Å, º)

**Table:**

*D*—H···*A*	*D*—H	H···*A*	*D*···*A*	*D*—H···*A*
C9—H9···O1	0.95	2.35	2.915 (6)	118
N1—H1···Cl1	0.87 (5)	2.32 (5)	3.177 (4)	167 (4)
C11—H11*A*···Cl1	0.99	2.75	3.541 (4)	137
C12—H12···Cl1^i^	0.95	2.73	3.530 (4)	143
C15—H15*B*···Cl1^i^	0.99	2.71	3.493 (5)	136
C11—H11*B*···Cl1^ii^	0.99	2.54	3.432 (5)	150
C13—H13···O1^iii^	0.95	2.54	3.066 (5)	115

Symmetry codes: (i) −*x*+1/2, −*y*+3/2, −*z*; (ii) *x*, *y*+1, *z*; (iii) −*x*+1/2, *y*−1/2, −*z*+1/2.
